# The challenges of predicting pesticide exposure of honey bees at landscape level

**DOI:** 10.1038/s41598-017-03467-5

**Published:** 2017-06-19

**Authors:** Noa Simon-Delso, Gilles San Martin, Etienne Bruneau, Christine Delcourt, Louis Hautier

**Affiliations:** 1 Beekeeping Research and Information Centre (CARI), Place Croix du Sud 1, Bte L7.04.01 1348, Louvain-la-Neuve, Belgium; 2 Walloon Agricultural Research Centre, Life Sciences Department, Plant Protection and Ecotoxicology Unit, Rue de Liroux, 2, B-5030 Gembloux, Belgium

## Abstract

To evaluate the risks of pesticides for pollinators, we must not only evaluate their toxicity but also understand how pollinators are exposed to these xenobiotics in the field. We focused on this last point and modeled honey bee exposure to pesticides at the landscape level. Pollen pellet samples (n = 60) from 40 Belgian apiaries were collected from late July to October 2011 and underwent palynological and pesticide residue analyses. Areas of various crops around each apiary were measured at 4 spatial scales. The most frequently detected pesticides were the fungicides boscalid (n = 19, 31.7%) and pyrimethanil (n = 10, 16.7%) and the insecticide dimethoate (n = 10, 16.7%). We were able to predict exposure probability for boscalid and dimethoate by using broad indicators of cropping intensity, but it remained difficult to identify the precise source of contamination (e.g. specific crops in which the use of the pesticide is authorized). For pyrimethanil, we were not able to build any convincing landscape model that could explain the contamination. Our results, combined with the late sampling period, strongly suggest that pesticides applied to crops unattractive to pollinators, and therefore considered of no risk for them, may be sources of exposure through weeds, drift to neighboring plants, or succeeding crops.

## Introduction

Pollinators like bees cover very large areas every day, visiting numerous plants for nectar, pollen, or gum collection and water sources. So doing, they also unintentionally collect airborne particles or substances diluted in the air. This has lead to using honey bees, a species often used as a model, and beekeeping products as biological indicators for environmental monitoring^1–18^. Monitoring of exposure to various environmental contaminants has already been carried out; these contaminants include heavy metals^2, 5, 14, 15, 17^, pesticides^3, 4, 11–13^, polycyclic aromatic hydrocarbons^6, 7, 9, 10, 18^ and radioactivity^1﻿6^. Unfortunately, it is often not possible to identify the specific sources of contamination.

The exposure of honey bees to pesticides has been linked to increased probability of colony disorders and losses^19–21^, alone or in combination with other stress-creating factors like poor nutrition or pathogen and parasite loads^22–24^. For this reason, it is crucial to understand the possible exposure pathways of honey bees to pesticides once they are released in the environment. Pesticide risk assessment is not just about the evaluation of the toxicity of the products. Ideally, we should also be able to accurately estimate how living organisms will be exposed to these products in the environment.

Efforts to model the exposure of bees to pesticides have been carried out recently for risk assessment purposes. Some models aim to estimate direct contact exposure for spray applications^25^, while others have focused on contact exposure through dust^26^ or on estimating pesticide intake^27–29^. Several routes of exposure are today aggregated for a more comprehensive estimation of the exposure of the honey bee colony^30^. However, more quantitative data on residue levels and their impacts on bee and colony are still needed^31^.

On the other hand, models of honey bee colony dynamics already integrate a number of stressors, and are a promising tool for impact evaluation of land management or stressors like pesticides at the landscape level^32, 33^. The quality of these models will depend on their capacity to predict the sources of contamination. The aim of this study is to test if it is feasible to identify the contamination origin by modeling the exposure probability at the landscape level.

We study the relationship between pesticide contamination of pollen pellets and both the botanical origin of the pollen and the areas of grasslands and different crops at four spatial scales around the apiaries (*n* = 40). Three pesticides with different physico-chemical properties are examined in detail as case studies. The results are interpreted relative to the authorized uses of these pesticides in the different crops present in the potential foraging area of honey bees. The aim here is therefore not to evaluate the toxicity of these pesticides or the consequence of the contamination on honey bee health, but to explore the contamination pathways and to evaluate the methodology on these case studies.

## Results

We consider three main data sets for the analyses: (1) pesticide load of pollen; (2) botanical origin of pollen and (3) landscape around the apiary.

### Pesticides

**Figure 1:**
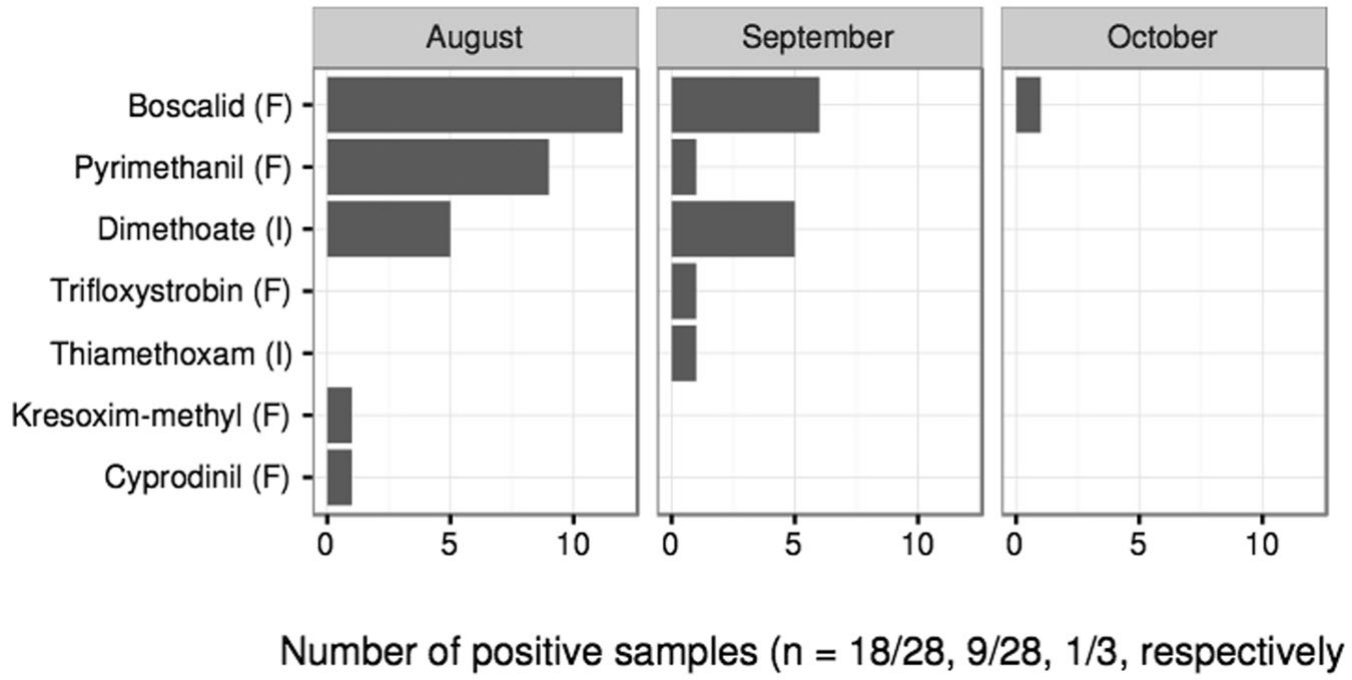
Frequency of pollen contamination per month and for each pesticide. Two samples from July are not shown. No pesticides were detected in these two samples. I = Insecticide, F = Fungicide.

About half of the analyzed samples contained at least one pesticide (n = 28/60). The most frequent were two fungicides, boscalid (n = 19, 31.7%) and pyrimethanil (n = 10, 16.7%), and one insecticide, dimethoate (n = 10, 16.7%). Boscalid residues ranged from 0.70 to 512 μg/kg, pyrimethanil residues ranged from 0.60 to 21.70 μg/kg and dimethoate residues ranged from 0.21 to 1.4 μg/kg (Supplementary Information 1 Table S1b). Four other active ingredients (a.i.) were detected with lower frequency (n = 1): trifloxystrobin, kresoxim-methyl, cyprodinil (fungicides) and thiamethoxam (a neonicotinoid insecticide) (Fig. 1). Eleven samples (18.3%) contained two or more a.i. simultaneously, reaching a maximum of three a.i. per sample and five a.i. per apiary. Pollen samples collected in July-August were more frequently contaminated than those from September-October (n = 18/29 and n = 10/31 respectively, binomial GLM, Likelihood Ratio (LR) = 5.7, df = 1, p = 0.017, Fig. 1). However, despite the small number of October samples analyzed, we were surprised to detect boscalid contaminations as late as 14 October.

### Pollen botanical origin

During the period considered (late July-October) the most abundantly collected pollen grains belonged to Brassicaceae, *Hedera elix* (ivy), *Trifolium* spp., *Phacelia tanacetifolia*, Rosaceae and Asteraceae (Supplementary Information 1 Fig. S2b). The August samples were characterized by more diversified pollen resources with predominance of *Trifolium* spp., Rosaceae and Asteraceae (including *Taraxacum* spp.) pollen. The pollen collected in September and October was less diversified and characterized by a higher abundance of ivy and *P*. *tanacetifolia* pollen (Supplementary Information 1 Fig. S2a). Brassicaceae pollen was found to be used during the whole period under review. These most abundant pollen types were also collected by 35/40 (87.5%) of the apiaries with the exception of Rosaceae (24/40, 60%) and *P*. *tanacetifolia* (12/40, 30%). This indicates that *P*. *tanacetifolia* fields are less present around the apiaries, but that they are massively visited by bees when they are present.

### Landscape description

**Figure 2:**
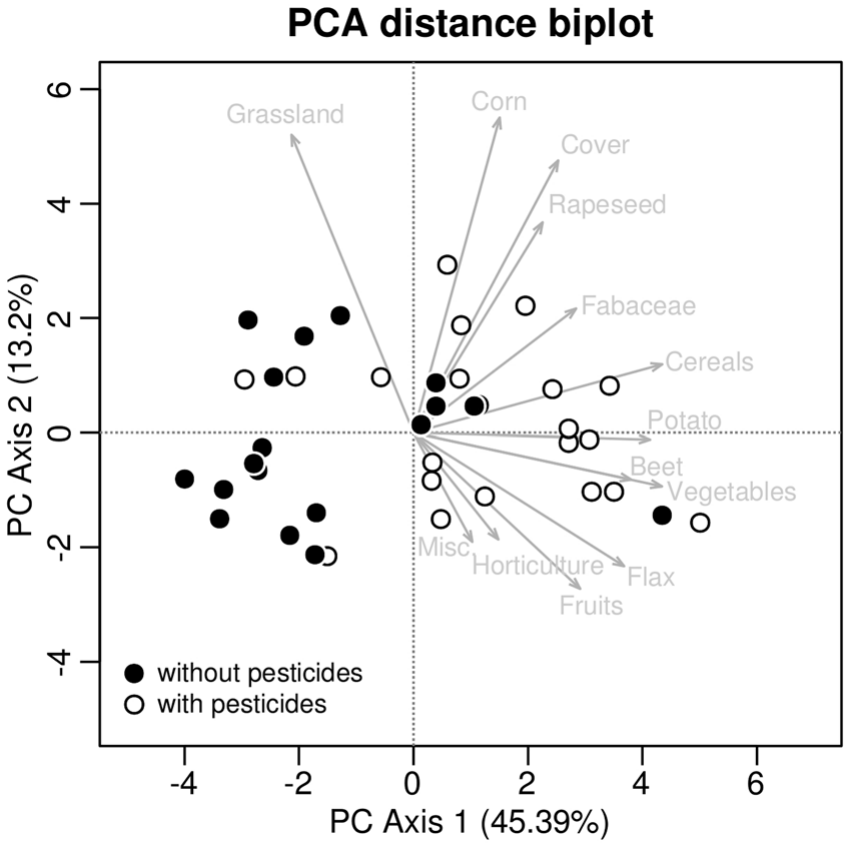
Principal Component Analysis distance biplot of the areas of crops and grasslands 3000 m around the apiaries. The areas were square root transformed and standardized before the analysis.

A high correlation exists between different crop areas (including grasslands), especially at the highest spatial scale (3000 m radius buffers) (Supplementary Information 1 Fig. S1c). These correlations are particularly high (0.78–0.93) for cereals, beet, potato and vegetables areas (Supplementary Information 1 Table S2a). Grassland areas are negatively correlated with most crop areas. The exploratory analysis showed a gradient in landscape composition around the apiaries: from landscapes dominated by crops to landscapes dominated by grasslands or with very little agricultural land use (urban or forest zones). The samples contaminated with pesticides are clearly more frequent in landscapes dominated by crops (Fig. 2). Cereals are present around all apiaries in 3000 m buffers. However, zooming into a radius of 500 m around the apiaries, one can divide the landscape into three groups: (1) dominance of cereals, beets and potatoes corresponding to the most intensive agricultural landscape; (2) dominance of cereals and grasslands (without beet and potato crops) corresponding to more extensive crop landscapes; and (3) areas dominated by grasslands and without cereals, beets and potatoes (clustering with heat map - see Supplementary Information 1 Fig. S1a and b).

### Case study 1 – Predicting bees’ exposure to boscalid

Boscalid is a carboxamid fungicide^34^ with systemic properties (octanol-water partition coefficient, log Kow = 2.96). In addition, this active substance is persistent in soil (DT50 typical = 200 days)^35^. In 2011, it was authorized in Belgium for the treatment of cereals, potatoes, rapeseed, many vegetables, fruits and horticulture.

We aimed to identify specific sources of boscalid contamination for bees. However, all crops have a predictive power with the exception of corn, rapeseed, cover crops and horticulture (AICc lower than the null model or with a difference <2, Table 1). The ten best models include as predictors: all crops and authorized crops areas at 3000 m, 1500 m and 1000 m and beet, potato and cereals at 3000 m (AICc differences > 18; Area Under the Receiver Operating Curve (AUC) 0.83–0.92). The models using 3000 m buffer data systematically have a better predictive performance, while the models using 500 m buffers systematically have the lowest one. Beet area in a 3000 m buffer is the best predictor of boscalid contamination despite the fact that the use of boscalid is not authorized in this crop. This predictor is four times better (ratio of AICc weights) than the second, using all crops as variable (i.e. areas of all crops combined) and more than 15 times better than the third, including potato and cereals areas in 3000 m (i.e. crops for which boscalid is authorized).

**Table 1:** Results of univariate Binomial GLMs modeling the probability that a pollen sample would be contaminated by a given pesticide vs the areas of different (groups of) crops and grasslands at different spatial scales (Buffer column, in meters).

Boscalid									
**ID**	**Buffer**	**Variable**	**AICc**	**Δ AICc**	**AICc w**	**AUC**	**Slope**	**LRT**	**p**
1	3000	Beet	40.01	0	0.604	0.894	0.33	29.14	<0.0001
2	3000	All Crops	42.81	2.798	0.149	0.894	0.18	26.34	<0.0001
3	3000	Authorized Crops	44.76	4.750	0.056	0.883	0.19	24.39	<0.0001
4	3000	Potato	45.24	5.231	0.044	0.920	0.36	23.90	<0.0001
5	3000	Cereals	45.74	5.734	0.034	0.863	0.21	23.40	<0.0001
6	1000	All Crops	46.06	6.054	0.029	0.846	0.47	23.08	<0.0001
7	1500	All Crops	46.40	6.386	0.025	0.851	0.31	22.75	<0.0001
8	1500	Authorized Crops	47.03	7.016	0.018	0.854	0.34	22.12	<0.0001
9	1000	Authorized Crops	47.35	7.344	0.015	0.840	0.53	21.79	<0.0001
10	500	All Crops	48.79	8.779	0.007	0.834	0.70	20.36	<0.0001
11–41	(…)								
42	—	NULL MODEL	66.92	26.91	0	0.500	—	—	—
43–55	(…)								
**Pyrimethanil**									
1	3000	Rapeseed	42.18	0	0.287	0.735	0.39	8.55	0.00346
2	1000	Rapeseed	43.69	1.515	0.134	0.756	0.61	7.03	0.00801
3	1500	Rapeseed	44.25	2.074	0.102	0.731	0.45	6.47	0.01095
4	3000	Flax	46.15	3.970	0.039	0.793	0.24	4.58	0.03240
5	3000	Horticulture	46.82	4.640	0.028	0.641	−1.93	3.91	0.04807
6	500	Beet	47.09	4.908	0.025	0.683	0.47	3.64	0.05643
7	3000	Cover	47.36	5.178	0.022	0.630	0.48	3.37	0.06643
8	500	Potato	47.36	5.184	0.021	0.659	0.46	3.36	0.06666
9	1000	Fabaceae	47.66	5.482	0.018	0.619	0.52	3.07	0.07999
10	3000	Cereals	47.78	5.599	0.017	0.719	0.07	2.95	0.08600
11–12	(…)								
13–55	—	NULL MODEL	48.5	6.322	0.012	0.500	—	—	—
14–55	(…)								
**Dimethoate**									
1	1000	Cereals	16.52	0	0.989	0.991	3.32	37.55	<0.0001
2	1000	All Crops	26.93	10.40	0.005	0.955	0.93	27.15	<0.0001
3	1500	Beet	29.61	13.08	0.001	0.942	0.77	24.47	<0.0001
4	1000	Beet	30.29	13.76	0.001	0.946	0.97	23.79	<0.0001
5	1500	Cereals	30.68	14.15	0.001	0.906	0.62	23.40	<0.0001
6	1500	Authorized Crops	31.37	14.85	0.001	0.920	0.67	22.70	<0.0001
7	1500	All Crops	31.4	14.88	0.001	0.929	0.46	22.67	<0.0001
8	1000	Authorized Crops	33.26	16.73	0	0.924	0.72	20.82	<0.0001
9	3000	Beet	33.73	17.21	0	0.915	0.36	20.34	<0.0001
10	3000	Authorized Crops	34.36	17.84	0	0.924	0.30	19.71	<0.0001
11–37	(…)								
38	NA	NULL MODEL	51.85	35.32	0	0.500	—		—
39–55	(…)								

Only the ten best models (lowest AICc) are shown along with the null model. LRT = Likelihood Ratio Test statistic (degrees of freedom = 1 for all models). Full tables available in the Supplementary Information 1.

**Figure 3:**
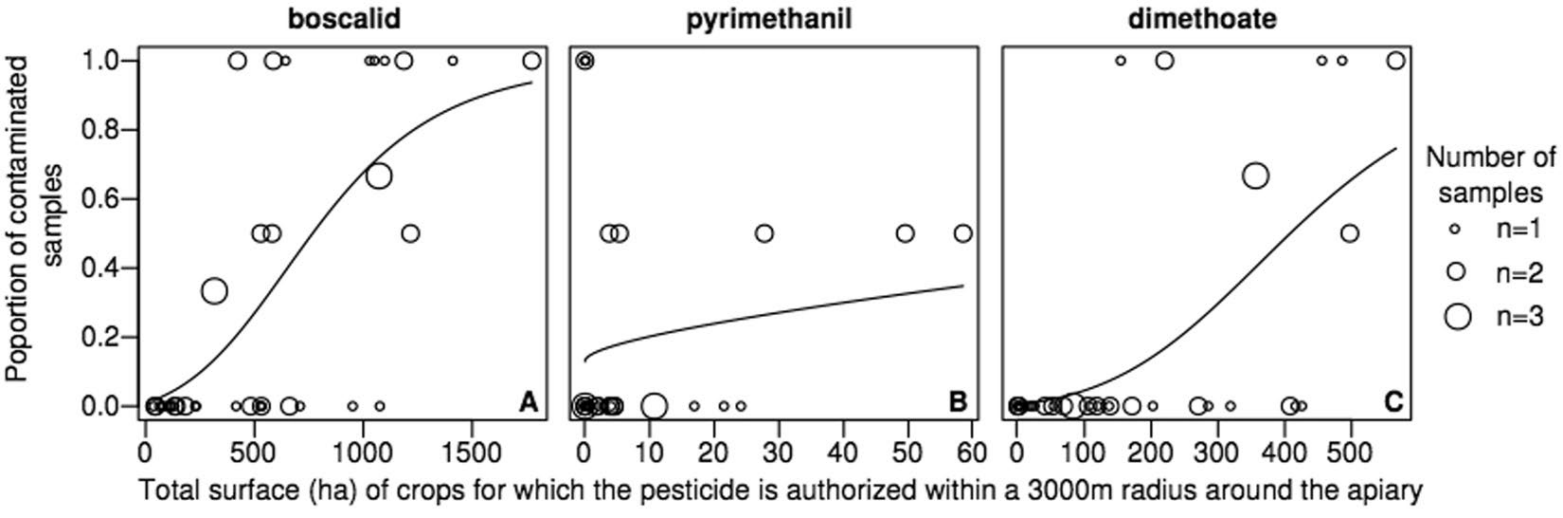
Observed proportion of samples contaminated for each pesticide and the corresponding predicted value (binomial GLM) relative to the areas of authorized crops around the apiary.

The predicted probability of boscalid contamination is close to 0 when no boscalid-authorized crops are present in a radius of 3000 m around the apiary and rises to 0.9 for areas of boscalid-authorized crops of 1500 ha (Fig. 3).

As for the botanical origin, Rosaceae and *P*. *tanacetifolia* pollen are systematically the most important predictors of boscalid contamination (AICc variable weight > 0.71- Table 2, positive relationship). There is no difference in boscalid contamination between July-August and September-October (AICc variable weight = 0.267).

**Table 2:** Results of the model selection for the GLMs modeling the presence of pesticides in the pollen vs the abundance of different pollen taxa.

	Boscalid				Pyrimethanil				Dimethoate		
	w	coef	se		w	coef	se		w	coef	se
*Intcpt*	1	−2.760	1.456	*Intcpt*	1	−4.179	2.274	*Intcpt*	1	−3.413	1.614
***ros***	**0**.**834**	**0**.**323**	**0**.**158**	***SepOct***	**0**.**959**	**−3**.**282**	**1**.**288**	***vic***	**0**.**996**	**0**.**822**	**0**.**269**
***pha***	**0**.**715**	**0**.**206**	**0**.**119**	***bra***	**0**.**952**	**0**.**747**	**0**.**341**	***pha***	**0**.**768**	**0**.**296**	**0**.**160**
*tar*	0.538	0.173	0.132	*ivy*	0.250	0.002	0.064	*ros*	0.388	0.109	0.116
*bal*	0.433	−0.108	0.102	*tar*	0.248	0.028	0.071	*tar*	0.280	0.054	0.087
*vic*	0.395	0.103	0.103	*api*	0.244	−0.021	0.067	*SepOct*	0.263	−0.124	0.374
*ivy*	0.350	0.062	0.071	*pha*	0.242	−0.014	0.045	*api*	0.261	−0.043	0.092
*bra*	0.332	0.056	0.066	*ast*	0.240	0.017	0.072	*ivy*	0.260	−0.027	0.061
*api*	0.273	0.039	0.067	*tri*	0.240	−0.016	0.065	*tri*	0.250	0.030	0.071
*SepOct*	0.267	−0.019	0.321	*ros*	0.237	0.004	0.048	*ast*	0.246	−0.029	0.084
*ast*	0.243	−0.004	0.058	*vic*	0.232	−0.011	0.068	*bra*	0.230	0.007	0.055
*tri*	0.231	0.002	0.042								

“w” = AICc variable weight, “coef” = models averaged coefficient, “se” = unconditional standard error. We interpreted only the explanatory variables with w > 0.60 (in bold). Intcpt = model intercept and SepOct = binary explanatory variable corresponding to the period: July/August or September/October. Abbreviation of the pollen types: api = Apiaceae, ast = Asteraceae, bal = Balsaminaceae, bra = Brassicaceae, ivy = *Hedera elix*, pha = *Phacelia tanacetifolia*, ros = Rosaceae, tar = *Taraxacum* spp., tri = *Trifolium*.

### Case study 2 – Predicting bees’ exposure to pyrimethanil

The second most frequently detected pesticide is pyrimethanil, a fungicide with systemic properties (log Kow = 2.84) and moderate persistence in soil (typical DT50 = 55 days)^36^. In 2011 it was authorized in Belgium for fruit production and horticulture (including plant nurseries and Christmas trees).

It was impossible to predict pyrimethanil contamination based on its authorized uses (Table 1). We even found contaminated pollen samples coming from apiaries with no crops in a radius of 3000 m for which pyrimethanil use is authorized (Fig. 3). The only crop that could predict the frequency of pyrimethanil contamination to a certain extent was rapeseed, for which pyrimethanil use was not authorized in Belgium in 2011 (AICc difference with the null model: 6.3–4.4). However, the AUCs of these rapeseed models are quite low (0.73–0.75), meaning that their discrimination capability is not very good with this dataset and should be even lower with a different testing dataset. Areas of flax and horticulture in a radius of 3000 m are weak predictors of pyrimethanil contamination (AICc difference 3.3 and 1.7, respectively). Pyrimethanil use is authorized for horticulture, but the model slope is negative, which means that we tend to observe less pyrimethanil contamination when the horticulture areas increase.

Among the models considering the pollen types, both the sampling period and the Brassicaceae pollen are good predictors of the presence of pyrimethanil (AICc variable weight > 0.95, Table 2). The contamination is significantly more frequent in July-August than in September-October. After controlling for the period, we find a strong positive relationship between pyrimethanil and the abundance of Brassicaceae pollen. This pollen probably comes from wild plants or cover crops like mustard, since rapeseed does not bloom at this time of the year in Belgium. Hence these pollen results (pyrimethanil-Brassicaceae) do not particularly support the landscape results (pyrimethanil-rapeseed).

### Case study 3 – Predicting bees’ exposure to dimethoate

Dimethoate is an organophosphorous insecticide, systemic (log Kow = 0.704), and non-persistent in soil (typical DT50 = 2.6 days)^37^. It was authorized in 2011 in Belgium to control insect pests in beets, peas, multi-annual fruit production, many vegetables, horticulture (incl. plant nurseries and Christmas trees), and until 2010 for potatoes.

Cereals, beet, all crops and authorized crops areas were the best predictors of pollen contamination by dimethoate (ten best models with differences of AICc relative to the null model between 35 and 17, AUC between 0.92 and 0.99, Table 1). Many other crop areas also had a good predictive power: vegetables, flax, grassland, horticulture, or potato. The models that consider the cropped areas at 1000 m and at 1500 m systematically had better predictive power, while the 500 m spatial scale systematically provided the worst models. Cereal areas in a 1000 m buffer were by far the best predictors of dimethoate contamination (AICc difference = 10.40), having a very high explanatory power (AUC = 0.99; AUC = 0.955 for the second best model).

As for the botanical origin, the probability of pollen contamination by dimethoate increases when pollen samples contain more *Vicia* spp. or *P*. *tanacetifolia* (AICc variable weight > 0.76, Table 2). There is no evidence for a difference of contamination between July-August and September-October.

## Discussion

Contamination of pollen pellets provide a representative image of flower contamination at a moment in time in contrast with the pollen that is stored in the beehive as beebread. The variety and frequency of pesticides detected in pollen pellets of our study is surprising for two reasons. Firstly, we did not expect pollen pellets to be contaminated with such a variety of pesticides at the latitude of Belgium and at this period of the year (late July-October) because few pesticides are applied so late in the season. Secondly, most of the crops with authorized uses for the detected pesticides do not bloom at this time of the year, which is confirmed by the botanical origin of pollen samples, containing mainly pollen from wild flowers and crops grown as cover or catch crops. As a result, the current approach of pesticide risk assessment and authorization based on the attractiveness to bees of different crops proves to be erroneous. Our results entail that pesticides applied to crops unattractive to bees as food sources like cereals or sugar beets can in fact be a source of exposure, either through weed contamination, drift or by the mobilization of residues of systemic/persistent products by succeeding crops. These results provide strong evidence that the concept of “crop not attractive to bees” is irrelevant to evaluating the risk of pesticide exposure. This conclusion is further supported by our landscape analyses. Our results add support to an increasing body of evidence indicating that pesticides applied to a crop are much more mobile than expected. Contamination of pollen pellets collected by bees late in the season, outside the period of pesticide application, has also been observed in other studies^21, 38, 39^, and contamination of wild flowers of the field margins has also been described^40–42^.

Simon-Delso *et al*.^19^ described pesticides found in beebread collected during the same period of the year as the present study in 21 apiaries. Boscalid, the most frequently detected molecule in pollen pellets (present study), was observed in beebread as the third most frequent residue. Pyrimethanil was reported in ten samples in pollen pellets, and was the sixth most common contaminant in beebread, after the fungicide iprodione and the synergist piperonyl butoxide. The insecticide dimethoate was detected in pollen pellets but not in beebread. Of the active ingredients detected only once, only trifloxystrobin was also found in beebread. These different contamination profiles indicate the value of analyzing different beekeeping matrices. Pesticides with low persistence like dimethoate are more likely to be found in pollen pellets than in beebread because they are collected at the beehive entrance, while beebread is pollen processed and stored within the colony during longer periods. However, this also indicates how difficult it is to characterize the whole range of pathways of pesticide exposure for honey bees.

There are long debates about the foraging radius of honey bees around their colony, most likely due to the variability of and dependence on the resources available in the surroundings, the weather conditions and the needs of the colony^43^. In our study, based on pollen samples collected from August to October, the models with best predictive value for pollen contamination were almost systematically those that considered crops located in a radius of more than 500 m, and up to 3000 m from the colony. Future work could test if differences in foraging range are translated into differences of pesticide exposure. However, the higher predictive power of 3000 m models may be independent from the foraging distance of bees because crop surfaces at higher landscape scales could just be a better indicator of agricultural practices in the close surroundings of the apiary (crops rotations, more/less intensive agriculture, etc.; see below).

We aimed to identify potential contemporaneous boscalid uses to explain direct pollen contamination. Boscalid-based products are authorized for a wide range of crops. They can still be used in orchards in August-September, a few weeks before the fruit harvest. They can also be used in August on beans. Consequently, direct exposure to treated crops like vegetables or orchards should be considered. However, the best predictor of boscalid contamination in pollen samples is sugar beet area in a radius of 3000 m, despite the fact that boscalid use is not authorized for this crop. Sugar beet is typically included in a crop rotation scheme with cereals and potatoes (sugar beet or potato/wheat/barley), in the most intensive agricultural areas of Belgium (Sandy-Loam Region). In contrast with cereals that are widely farmed even in less intensive areas, sugar beet may be a better indicator of cereals farmed in a more intensive way and hence potentially receiving more pesticides. After the beet model, all crops is the model with best predictive value and the most frequently found among the ten most predictive models (at all four landscape scales), followed by authorized crops. This, together with the fact that individual crops (i.e. potatoes and cereals) complete the list of the ten most predictive models, indicates that direct exposure to treated crops is unlikely to be the only source of contamination, as pesticides containing boscalid are typically applied to these crops much earlier in the season. Furthermore, with the exception of beans, none of the crops for which boscalid is authorized are attractive to honey bees for food collection at this time of the year. The high persistence of the molecule could possibly explain contamination later in the year or even during the next year. This may lead to the contamination of wild flowers or of succeeding crops like cover/catch crops. Contamination linked to cover/catch crops is supported by the positive relationship between boscalid presence and the abundance of *P*. *tanacetifolia* pollen observed in this study. *P*. *tanacetifolia* is not native in Belgium and is only used as a catch crop and rarely in gardens. Other persistent and systemic pesticides (e.g. neonicotinoids) can contaminate wild flowers in field margins^41, 42^ and succeeding crops^44–47^.

The case of pyrimethanil pollen contamination is difficult, because none of the authorized uses predict its presence. In addition, we found a number of positive samples (n = 4) with no crops for which this pesticide was authorized in a radius of 3000 m around the corresponding apiary. At this point, we considered the following hypotheses: (1) the contamination came from further than 3000 m; (2) the notification of crop areas for which this fungicide is authorized is not complete (e.g. Christmas trees, some horticultural or vegetable crops); (3) there is an illegal use of pyrimethanil in rapeseed earlier in the season (rapeseed is already harvested at this time of the year but the product is moderately persistent). The later hypothesis seems unlikely because there are many other efficient fungicides authorized for rapeseed and the model discriminatory power was quite low for this crop. Pyrimethanil-based products are also used in orchards at the beginning of the season, but we found no support for a pyrimethanil-Fruit areas relationship. These products could also be used for the production of some specific vegetables like peas, beans, and other legumes. However, in the year of the study, 2011, these uses were not authorized in Belgium. Pea fields that are typically harvested in July could be followed by mustard as cover/catch crop. This would match the link we found between the residues of pyrimethanil and the pollen of Brassicaceae in August. Models specifically using areas of cultivated peas and broad beans (instead of the grouped category fabaceae) showed better predictive value at 1500 m and 1000 m (difference of AICc = 3.60 and 2.85, Likelihood Ratio Test statistic (LRT p values = 0.016 and 0.024), but their discrimination power was not very high (AUC = 0.67 and 0.68 - see Supplementary Information 1 Table S6 for details). As a result, this hypothesis remains only a putative scenario that should be tested by specific sampling on peas in the field.

Dimethoate is not a persistent pesticide. We can therefore assume that pollen contamination came from an application during August-September. The best predictors of the presence of dimethoate in pollen were cereals (non-authorized use and crop already harvested at this time of the year), beet (authorized use, but unlikely to be applied because there are no insect pests at this time of the year), and all crops. At this time of the year, vegetables (e.g. carrots and Brussels sprouts) are the only crops possibly being treated with dimethoate for which the area can be used to predict the frequency of dimethoate in our models. As a result, the fact that cereals or sugar beets are the best predictors for dimethoate contamination could be because these large arable crops are good indicators of intensive, large scale, vegetable production, which may be included in crop rotation schemes: there is a strong correlation (R > 0.77) between vegetables and beet, cereals and potato areas (Supplementary Information 1 Fig. S1c). However, none of these vegetable crops are in bloom at the sampled period (except occasionally carrots), which made us wonder about the pertinence of this hypothesis. The palynological results show that pollen from *Vicia* spp. and *P*. *tanacetifolia* are positively linked with the presence of dimethoate. It is common agricultural practice in the region to include flowering strips in the borders of vegetable fields. Therefore, a possible explanation could be that *Vicia* spp. occur in field margins or that *P*. *tanacetifolia* is planted in flowering strips and that their flowers get contaminated by drift with dimethoate applied on the field. The abundance of *Vicia* spp. pollen is positively correlated to vegetables, beet, potato and cereals areas (see Supplementary Information 1 Fig. S3).

In conclusion, our findings show that the highest spatial scales (3 km) provide the best predictive power for pollen contamination. Pesticides applied to “non-bee-attractive” crops like cereals or sugar beets, generally considered of negligible risk for bees, can in fact be a source of exposure through weeds, through drift to neighboring plants or through succeeding crops. These results imply that the concept of “bee-attractive crop” (i.e. a crop visited by bees for nectar and/or pollen collection) is irrelevant for risk assessment and should not be used as a criterion for pesticide authorization. At the landscape level, honey bee exposure to pesticides depends on pesticide use level, physicochemical characteristics, period of the year and landscape composition. Our findings show that the task of modeling the exposure of bees to pesticides once released in the environment may be more complicated than expected. We were able to efficiently predict exposure for two pesticides by using very broad indicators of cropping intensity, but it remains difficult to track the direct source of contamination in the landscape. For the third pesticide, we were not able to find any convincing landscape model that could explain the contamination. On the other hand, our results have consequences for policies and agricultural practices intended to promote the multiplication of nutritional resources for pollinators, like flowering strips, buffer zones, catch crops with melliferous flowers, etc. These should be designed and applied in parallel to policies and practices leading to pesticide use reduction i.e. integrated pest management, organic farming or agro-ecological practices, precision farming and favoring non-persistent/non-systemic pesticide active ingredients. Without such considerations, instead of favoring pollinators through habitat improvement or food availability, we may transform these areas into highly risky zones or even ecological traps for pollinators.

## Methods

### Field work – sample collection

A group of voluntary beekeepers were requested to participate in the study, with a total of 40 apiaries. Pollen samples (n = 80) were taken from two random colonies per apiary with the help of a PVC pollen trap (Nicot®), placed during one or two days to collect a minimum of 20 g of pollen pellets. Samples from both colonies were pooled together. The samples were collected once, twice or four times per month from mid-July to mid-October 2011. Most of the pollen samples were collected in August and September 2011 (n = 32 and 36 respectively). Two pollen samples were collected in July and ten samples in October. The July samples were taken on 24 and 30 July, and were similar to the August samples in terms of botanical origin of the pollen (Supplementary Information 2 at https://figshare.com/s/86785808b5709331aa1c). One sample collected in April 2012 was removed from the dataset before analysis because this unique sample had a completely different pollen composition and was not comparable to the other samples. Samples were placed in hermetic plastic bags and stored at −20 °C until analysis.

### Sample processing

Samples collected from the same apiary during the same month were pooled together and thoroughly mixed. One gram of the blend was sampled for palynological analysis. Whenever the sample quantity allowed it, at least 42 grams of the monthly blend were shipped for pesticide residue analysis (n = 60). Frozen samples were sent for pesticide analysis in dry ice.

### Pesticide analyses

The monthly samples of pollen pellets were sent to Floramo Corporation, Italy. A multi-residue analysis was used based on the methodology described by Wiest *et al*.^48^, and 45 pesticides/metabolites were analyzed in pollen pellets (Supplementary Information 1 Table S1). The extraction method was based on a modified “QuEChERS method”: two-step Solid/Liquid extraction with solvent and MSPD (Matrix Solid Phase Dispersion) purification as follows: 10 g of the pollen sample extracted with acetonitrile/water followed by liquid/liquid purification with hexane and combined with MSPD purification on PSA and salts. Finally, the purified extract was concentrated below 100 µl and injected into UPLC-MS/MS (Ultra Pressure Liquid Chromatography coupled with tandem mass spectrometry) and gas chromatography coupled with tandem mass spectrometry (GC-MS/MS) programmed in MRM (Multiple Reaction Monitor) mode with two transitions/a.i. Pesticide analysis was possible for 28 samples from August and September each, 1 sample from July and 3 samples from October.

### Palynological analyses

The extraction and homogenization method was inspired by the harmonized method of pollen analysis with acetolysis developed by Erdtman^49^. A minimum of 1000 pollen grains were counted and identified per slide^50^ at 500x microscopic magnification as described by von der Ohe *et al*.^51^. Pollen grains were generally identified up to their taxonomical family due to the difficulty of differentiating plant species. In a few easy cases, identification was performed up to genus level (*Taraxacum* spp., *Trifolium* spp., etc.) and to species level for ivy (*Hedera helix*) and lacy phacelia (*Phacelia tanacetifolia*).

### Landscape data

We measured the areas of different kinds of detailed agricultural land use (i.e. different crops and grasslands) in a circle (buffer) with a radius of 500, 1000, 1500 and 3000 m around the 40 apiaries. We used the official Land Parcel Identification System (SIGEC) used by the Walloon administration to distribute agricultural subsidies to the farmers. This land use information was not available for one of the apiaries located outside the Walloon region. These detailed land use categories (n > 50) were pooled into thirteen more general categories (“crops”) according to their agronomic similarities (see Supplementary Information 1 Table S2b). For each of the detailed land use categories, we determined whether the three most frequent pesticides observed in this study could be used by checking the official pesticide use authorizations in Belgium in 2011. This allowed us to calculate the areas of authorized crops for each of these pesticides around the apiaries (see Supplementary Information 1 Table S2a).

### Statistical analyses

All analyses were performed in R^52^. All raw datasets and R scripts are provided as supplementary information (Supplementary Information 2 at https://figshare.com/s/86785808b5709331aa1c). For confidentiality reasons, we are not allowed to share the exact location of the apiaries nor the agricultural land use geodata.

We considered three main datasets for the analyses: pesticides, landscape (areas of different crops and grasslands) and pollen (taxonomical origin). For the pesticides we used only presence/absence information in each pollen sample. The pollen samples had a variable number of total pollen grains (around 1000). To make direct comparison possible, the number of pollen grain (x) was standardized as x * 1000/N (N = total grain number in the sample) and rounded to units.

### Predicting bees’ exposure to different pesticides with crops and grassland areas

As crop areas are only available at the apiary level, we used binomial GLMs with the proportion of positive samples in a given apiary as the response variable for each pesticide. Preliminary analyses showed that the crop areas are strongly correlated, causing multicollinearity problems when they are used as predictors in multiple regression approaches. Grouping correlated predictors was not an option here because we wanted to keep them separated to interpret the results relative to the authorizations of pesticide use for each crop. Consequently, we decided to use binomial GLMs with only one explanatory variable at a time. We built separate univariate models for each grouped land use surface and for each of the four spatial scales. We also used two additional predictors (at four scales): 1) all crops: sum of the crop areas i.e. without grasslands and without taking into account the product authorizations; 2) authorized crops: sum of the crop areas for which the product is authorized. A “null model” was also built with no explanatory variable, i.e. this model estimates the mean proportion of samples contaminated in the dataset. All areas were square root transformed, because this improved the quality of the models (i.e. linearity and homogeneity of the residuals). At the lowest spatial scales (500 m and 1000 m) some of the minor crops were totally absent from all apiaries and the corresponding models were therefore not estimated.

These models were compared in terms of AICc and AICc weights^53^ between each other and more particularly with the “null model”. Models with lower AICc are considered to be better (good fit but not overly complex to allow extrapolation to other datasets), and a difference of AIC lower than 2 is often considered as negligible. In addition we computed for each single model a likelihood ratio test which compares the model to the null model. We also computed the Area Under the Receiver Operating Curve (AUC or AUROC) as a descriptive statistic of the capacity of each model to discriminate (in this dataset) between apiaries with the pesticide (frequency in the samples > 0) or without it (frequency = 0). An AUC = 1 indicates a perfect discrimination capability (all predicted presences are effective and none of the predicted presences are absences). If AUC = 0.5, the model predictions are as good as pure chance.

We checked the spatial correlation of the best model residuals for each pesticide with a spline correlogram. Spatial correlation was always low and not significantly different from 0.

### Predicting bees’ exposure to different pesticides with pollen composition

The pollen data are available at the sample level and there were no multicollinearity problems with these data. Consequently, we computed binomial GLMs with the presence/absence of the pesticide in the sample as response variable and the ten most common pollen types as explanatory variables. We also added the period of the year (July-August or September-October) as explanatory variable. The pollen data were log(x + 1) transformed because this clearly improved the model fit. With 59 pollen samples from 39 apiaries, some pollen samples came from the same apiary and were therefore not independent. We first tried to use binomial Generalized Linear Mixed Models with the apiary as random effect to take this pseudo-replication into account, but most of these models did not converge, probably because most of the apiaries in the sample had only one or two replicates (rarely three). Our results with the simple GLMs are therefore probably slightly anti-conservative. We removed the explanatory variable Balsaminaceae because none of the pollen samples containing grains of this family were contaminated with dimethoate. This lack of variability posed problems in the statistical analyses.

For each pesticide, we computed models for all possible combinations of explanatory variables (2048 models) along with their AICc and AICc model weight. The AICc model weight was used to compute shrinkage model averaged coefficients, unconditional standard errors and AICc variable weights^53^. The AICc model weight is a measure of model selection uncertainty (probability that a model will have the lowest AICc if we resample the data, given a set of models). The AICc variable weights allow us to compare the relative importance of the explanatory variables (it gives the probability that a given variable will be in the best - lowest AICc - model if we resample the data). We interpreted only the explanatory variables with an AICc variable weight > 0.6. The model averaged regression coefficients are shrunk toward 0 when the corresponding explanatory variable is present only in “bad” models.

### Data availability

Supplementary information is available in a public repository at https://figshare.com/s/86785﻿808b5709331aa1c

## Electronic supplementary material

Supplementary information
