# Supplementary material for: The challenges of predicting pesticide exposure of honey bees at landscape level
Source: Sci Rep. 2017 Jun 19;7:3801. doi: 10.1038/s41598-017-03467-5 (PMC5476569; doi:10.1038/s41598-017-03467-5)

Supplementary figures and tables for the paper

“The challenges of predicting pesticide exposure of honey bees at landscape level"

Noa Simon-Delso[a*], Gilles San Martin[b], Etienne Bruneau[a], Christine Delcourt[a], Louis Hautier[b]

[a] Beekeeping Research and Information Centre (CARI), Place Croix du Sud 4, 1348 Louvain la Neuve, Belgium
[b] Walloon Agricultural Research Centre, Life Sciences Department, Plant Protection and Ecotoxicology Unit, Rue de Liroux, 2, B-5030 Gembloux, Belgium
*Corresponding author: Noa Simon-Delso, simon@cari.be

Contents

Introduction **2**
Pesticides: exploratory analysis **2**
Table S1a: Pesticides analyzed + LOQ 2
Table S1b: Summary table of the pesticides found in pollen samples 4
Agricultural landscape: exploratory analysis **5**
Table S2a: Landscape land use categories used 5
Table S2b. Total areas (ha) for each grouped agricultural land use category in the 3000 m buffers around the apiaries and the areas of authorized crops for each pesticide 7
Fig. S1a and S1b : Heatmaps of the landscape dataset 7
Fig S1c. Heatmap of the correlation matrix of the areas of crops and grasslands 3000 m around the apiaries 10
Pollen: exploratory analysis **11**
Table S3: Pollen codes 11
Fig. S2a: Heatmap of the pollen dataset 12
Fig. S2b: Total number of pollen grains from different botanical origin per month 14
Pesticides vs Agricultural Landscape **15**
Boscalid 15
  Table S4: Boscalid vs cultures models 15
Pyrimethanil 18
  Table S5: Pyrimethanil vs cultures models 18
  Table S6: Pyrimethanil vs peas crops models 20
Dimethoate 21
  Table S7: Dimethoate vs cultures models 21
Pollen vs Landscape **23**
Fig. S3 Pollen vs landscape RDA triplot 23

Introduction

Please look at the Supplementary Material 2 for full analysis with code, data and full results and interpretation (including these figures and tables).

Pesticides: exploratory analysis

Table S1a: Pesticides analyzed + LOQ

Summary of all pesticides (and some products of degradation) that have been looked for into the trap pollen samples + LOQ values in µg/kg (10^-3 mg/kg)

fullname	type	LOQ_Pollen
Amitraz	A	5
Beta-cyfluthrin	I	5
Bifenthrin	I	3
Boscalid	F	7.5
Captan	F	7.5
Carbaryl	I	0.75
Chlorpyriphos	I	5
Clothianidin	I	0.75
Coumaphos	A	5
Cyfluthrin	I	5
Cyprodinil	F	5
Deltamethrine	I	5
Dimethoate	I	0.75
Esfenvalerate	I	7.5
Fenpropimorph	F	7.5
Fipronil	I	1
Heptenophos	I	7.5
Imidacloprid	I	0.75
Indoxacarb	I	7.5
Iprodione	F	7.5
Kresoxim-methyl	F	7.5
Lambda-cyhalothrin	I	7.2
Piperonyl butoxide	S	7.5
Pirimicarb	I	5
Propamocarb	F	5

fullname	type	LOQ_Pollen
Pyraclostrobin	F	5
Pyrimethanil	F	5
Tau-fluvalinate	IA	7.5
Tebuconazole	F	7.5
Tebufenozide	I	7.5
Terbuthylazine	H	7.5
Thiacloprid	I	0.75
Thiamethoxam	I	0.75
Thiophanate-methyl	F	5
Trifloxystrobin	F	7.5
Zoxamide	F	7.5
Fipronil carboxamide	I	7.5
Fipronil desulfinil	I	7.5
Fipronil sulfone	I	7.5
5-OH Imidacloprid	I	7.5
Desnitroimidacloprid	I	7.5
6-Cloronicotinc acid	I	7.5
Olefin	I	7.5
Urea Derivate	I	7.5

Table S1b: Summary table of the pesticides found in pollen samples

Type : F = Fungicide, I = Insecticide. Nb = number of positive samples. NbTot = total number of samples analyzed. The columns Min, Max, Median, Mean and SD (Standard deviation) provide descriptive statistics of the quantities (mg/kg) observed in the positive samples only.

Pesticide	Type	Nb	NbTot	Min	Max	Median	Mean	SD
boscalid	F	19	60	0.70	512.00	4.10	37.93	116.50
pyrimethanil	F	10	60	0.60	21.70	1.90	4.93	6.60
dimethoate	I	10	60	0.21	1.40	0.40	0.53	0.40
trifloxystrobin	F	1	60			15.40		
thiamethoxam	I	1	60			1.60		
kresoxim-methyl	F	1	60			2.40		
cyprodinil	F	1	60			1.50		

Agricultural landscape: exploratory analysis

Table S2a: Landscape land use categories used

In the following table we show how we have used the different agricultural land use informations that were available :

- Agri : is the detailed land use types provided by the SIGEC (agricultural aids administration). The name are in French but most of them have been translated in the paper.
- Agri_group : we have grouped the detailed Agri into these more general denominations to reduce the number of categoriesAreas : sum of the areas (ha) in the 3000 m buffers for all the apiaries
- Dime, Pyri, Bosc : is the use of dimethoate, pyrimethanil, boscalid authorized at least partially for each detailed culture type ? When we have calculated the areas of cultures authorized for each pesticide, we used the detailed Agri codes before grouping. For example for dimethoate, the authorized Fabaceae areas are only using the Peas areas, not the other detailed cultures that have been grouped under the name of Fabaceae for other analyses.

Agri	Agri_group	Areas	Bosc	Dime	Pyri
BoscBetterave sucrière	Beet	4578	yes		
Chicorée à inuline	Beet	675	yes		
Betterave fourragère	Beet	63.7	yes		
Chicorée à café	Beet	3.4	yes		
Froment d'hiver	Cereals	12986			yes
Orge d'hiver	Cereals	3004			yes
Epeautre	Cereals	600			yes
Froment de printemps ou froment alternatif	Cereals	287.5			yes
Avoine	Cereals	151.4			yes
Triticale	Cereals	101.5			yes
Orge de printemps	Cereals	74			yes
Autres grains (p.e mélanges)	Cereals	62.7			
Orge de brasserie	Cereals	26.8			yes
Autres céréales (Sorgho, millet, alpiste et blé dur)	Cereals	8.6			
Seigle d'hiver	Cereals	0.9			yes
Maïs ensilage	Corn	3976			
Maïs grain	Corn	696.2			
Tournière enherbée	Cover	565.5			
Autres couvertures (Moutarde, Phacélie,etc.)	Cover	47			
Autres couvertures dont "mélange certifié"	Cover	18.6			
Pois (autres que récoltés secs)	Fabaceae	390.2	yes		

Agri	Agri_group	Areas	Bosc	Dime	Pyri
Luzerne	Fabaceae	161.2			
Mélange protéagineux (culture principale) + céréales	Fabaceae	95.2			
Fèves et féveroles (sec)	Fabaceae	30			
Mélange graminées et légumineuses	Fabaceae	25.4			
Trèfle	Fabaceae	24			
Pois (sec)	Fabaceae	22			
Légumineuses	Fabaceae	0.1			
Lin textile	Flax	599.1			
Lin oléagineux	Flax	4			
Cultures fruitières pluriannuelles	Fruits	212.8	yes	yes	yes
Cultures fruitières annuelles	Fruits	11.7		yes	yes
Vignes	Fruits	0.9		yes	yes
Prairie permanente	Grassland	15902			
Prairie temporaire	Grassland	1371			
Pâturage à statut particulier	Grassland	52.5			
Cultures horticoles non comestibles	Horticulture	15	yes	yes	yes
Sapins de Noël	Horticulture	5	yes	yes	
Pépinières	Horticulture	4.9	yes	yes	
Autres fourrages	Miscellaneous	428.2			
Couvert naturel	Miscellaneous	37			
Boisement	Miscellaneous	11.6			
Miscanthus	Miscellaneous	7.4			
Terres retirées de la production	Miscellaneous	7.3			
Sarrasin	Miscellaneous	1.8			
Chanvre non-alimentaire autre que chanvre textile	Miscellaneous	0.1			
Pomme de terre de consommation	Potato	2734			yes
Pomme de terre (Plants)	Potato	114.6			yes
Pomme de terre féculière	Potato	31.7			yes
Pomme de terre (arrachage avant le 1er aout)	Potato	8.8			yes
Pomme de terre (primeur, arrachage avant le 20 juin)	Potato	0.2			yes

Agri	Agri_group	Areas	Bosc	Dime	Pyri
Colza et navette d'hiver	Rapeseed	941.5			yes
Colza et navette de printemps	Rapeseed	11.5			yes
Cultures maraîchères	Vegetables	587.7	yes		yes
Cultures maraîchères sous verre	Vegetables	1.8			
Plantes aromatiques	Vegetables	0.1			yes

Table S2b. Total areas (ha) for each grouped agricultural land use category in the 3000 m buffers around the apiaries and the areas of authorized crops for each pesticide

The small differences between the areas for a same grouped land use are due to the fact that we considered the authorizations of each product relative to the detailed land uses.

Grouped land use	Total	Bosc.	Pyrim.	Dimet.	Detailed land uses
**Cereals**	16695	16632	0	0	wheat, barley, spelt, oat, triticale, etc.
**Grassland**	15993	0	0	0	permanent and temporary grasslands
**Beet**	5190	0	0	5190	sugar and fodder beet, root chicory
**Corn**	4211	0	0	0	grain and fodder corn
**Potato**	2711	2711	0	0	mainly potato for food
**Rapeseed**	947	947	0	0	rapeseed (mainly sown before the winter)
**Fabaceae**	727	0	0	386	peas, alfalfa, mixed protein crops, etc.
**Cover**	611	0	0	0	cover crops and set-aside field borders
**Flax**	593	0	0	0	flax
**Vegetables**	521	520	0	520	vegetables (including glasshouses)
**Miscellaneous**	456	0	0	0	misc. land uses, mainly "other fodder"
**Fruits**	225	225	225	213	perennial and annual fruits, vineyards
**Horticulture**	25	15	25	25	plant and tree nurseries, Christmas trees

Fig. S1a and S1b : Heatmaps of the landscape dataset

Methods

The row dendrogram is based on Euclidean distance. The column dendrogram is based directly on the correlation matrix as similarity matrix. Both row and columns dendrogram use the Ward agglomerative algorithm. The blue colored band close to the row dendrogram represent the number of different pesticides for each apiary (white = 0, deepest blue = 5). The numbers represent the surface in hectares.

Fig. S1a 500 m buffer heatmap

The 500 m buffer heatmap is quite informative. You can clearly see that there are 3 groups of sites. One group with beet and potato cultures (on top), one group without beet and potato cultures but with cereals (in the middle) and one group with very few crops and generally more grasslands (at the bottom + 2 sites without major crops and without grasslands probably more urbanized areas)

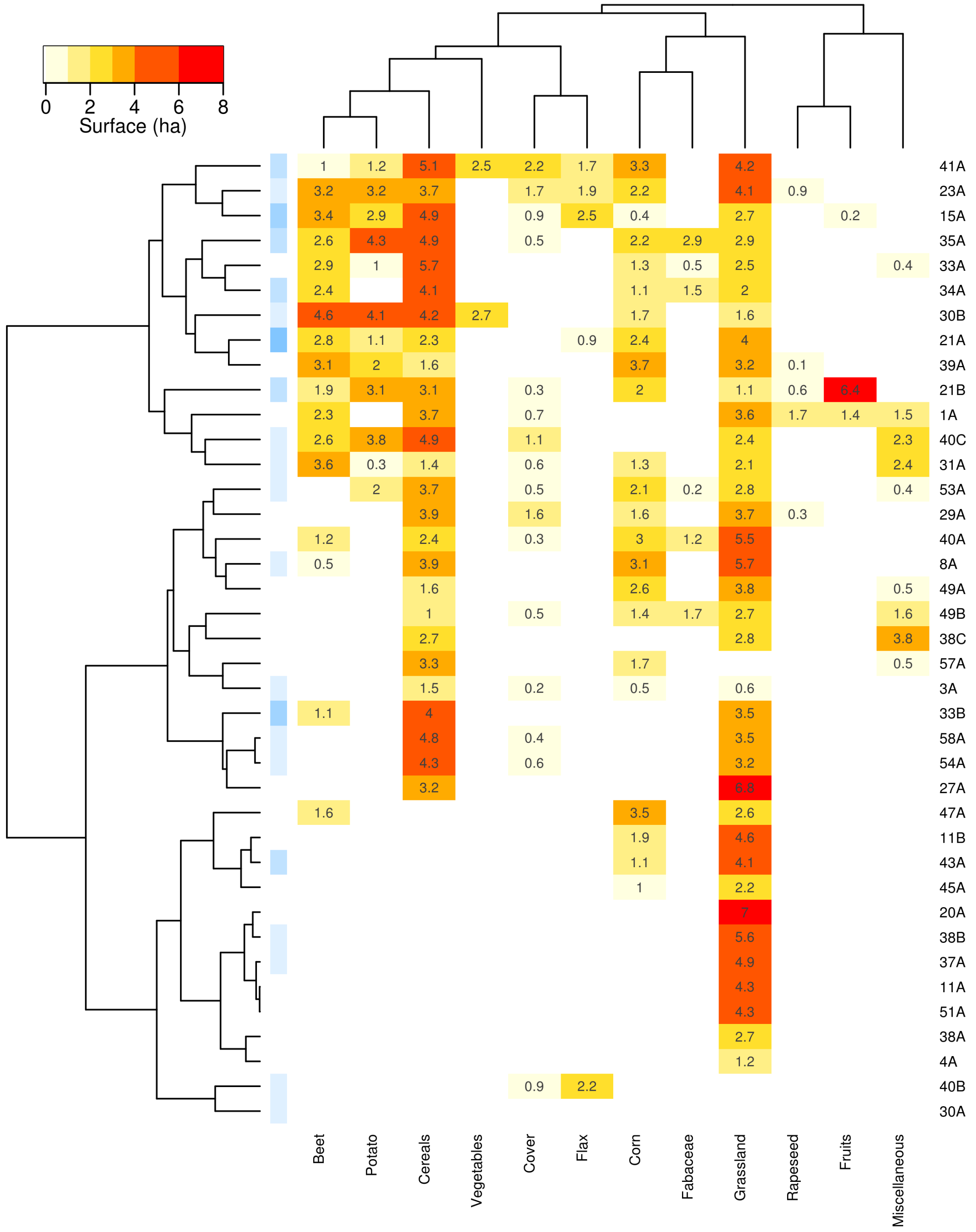

Fig. S1b 3000 m buffer heatmap

The 3000 m heatmap is less structured. You can see 2 clear groups : one with few big crops at the bottom (with or without grasslands) and one with lots of crops on top, particularly beet, potatoes, vegetables, etc. In fact you have a clear gradient in the dataset from sites with a lot of crops toward sites with few crops (either with grasslands or with non agricultural land uses). You can see also quite clearly that the samples with pesticides are clearly in the second group.

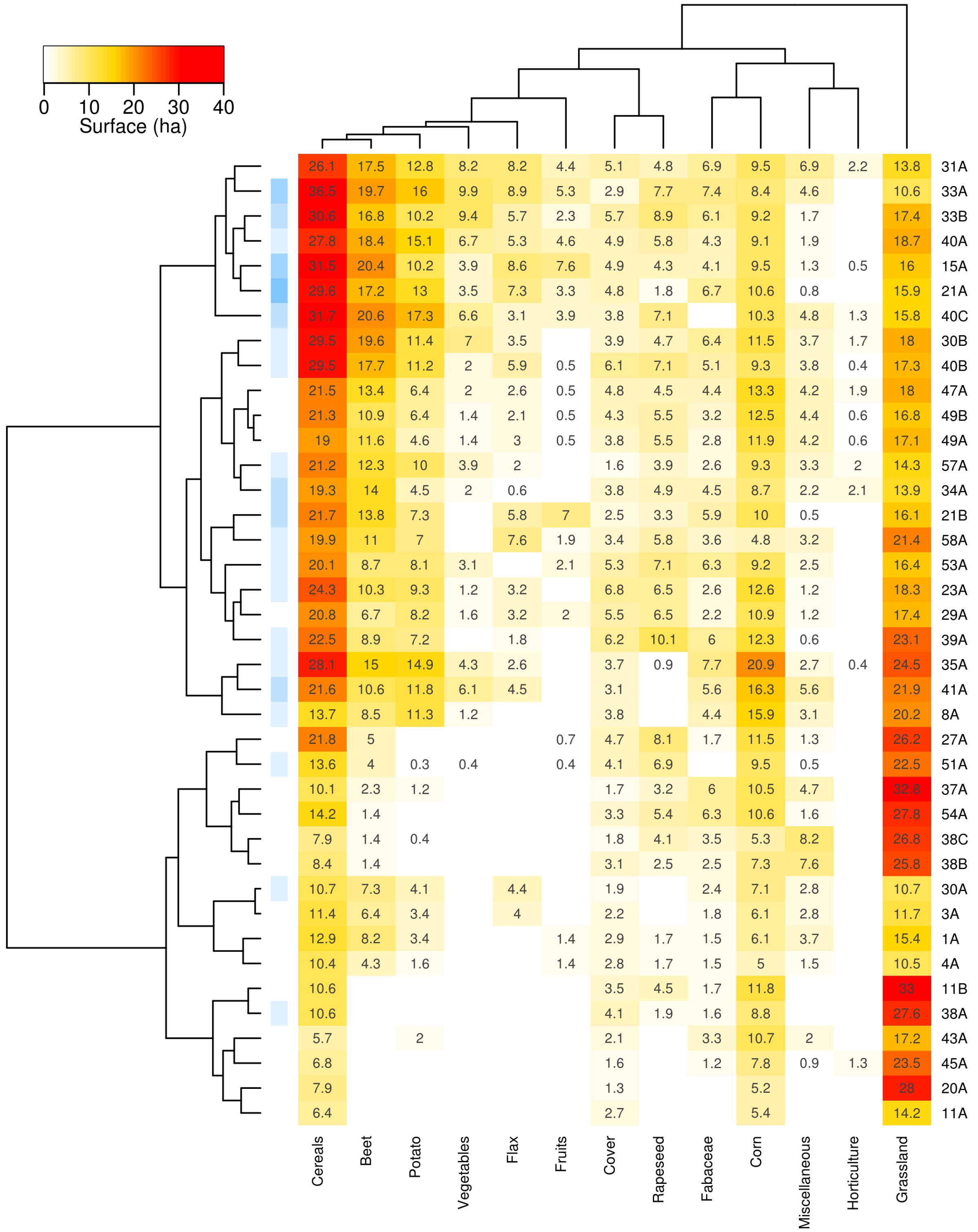

Fig S1c. Heatmap of the correlation matrix of the areas of crops and grasslands 3000 m around the apiaries

The numbers represent the Pearson correlation * 100. The dendrograms are build with a ward algorithm on the correlation matrix transformed into distance matrix.

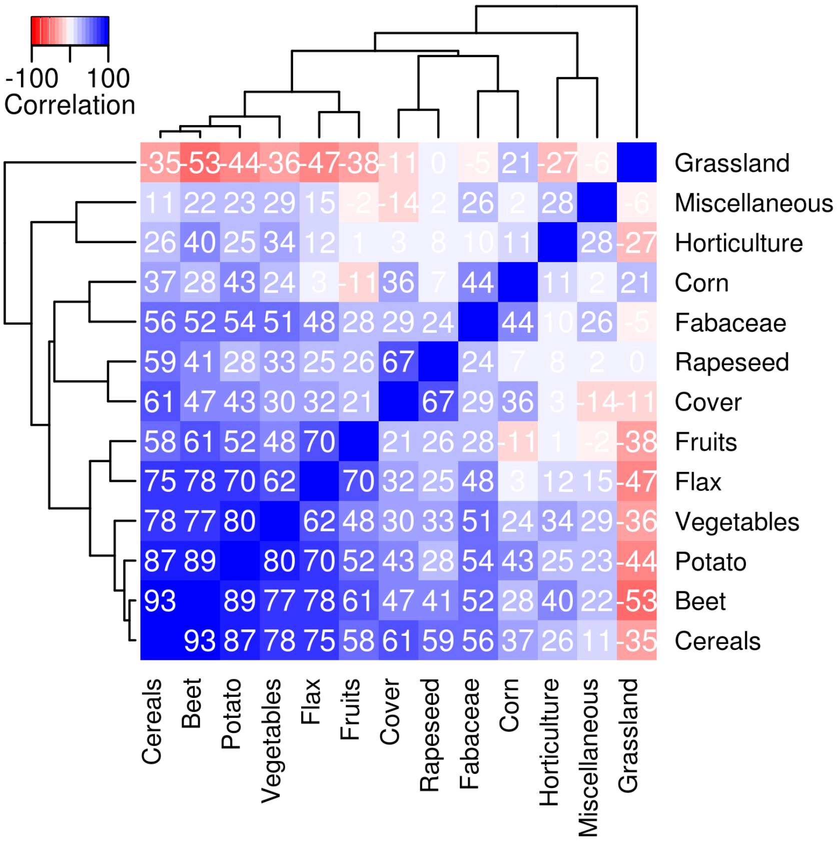

Pollen: exploratory analysis

Table S3: Pollen codes

The pollen codes are summarized in the following table :

	Pollen_type	Pollen_code
**1**	Anacardiaceae	ana
**2**	Apiaceae	api
**3**	Asteraceae	ast
**5**	Balsaminaceae	bal
**6**	Borraginaceae	bor
**7**	Brassicaceae	bra
**8**	Campanulaceae	cam
**10**	Caprifoliaceae	cap
**11**	Centaurea	cen
**13**	Cereals	cer
**14**	Chenopodiaceae	che
**15**	Cistaceae	cis
**18**	Epilobium	epi
**19**	Ericaceae	eri
**21**	Fruit trees	fru
**22**	Lamiaceae	lam
**23**	Hedera elix	ivy
**24**	unknown	unk
**25**	Oleaceae	ole
**26**	Sedum	sed
**27**	Rumex	rum
**28**	Phacelia	pha
**30**	Taraxacum	tar
**31**	Plantago	pla
**32**	Poaceae	poa
**35**	Renonculaceae	ren
**37**	Rhamnaceae sl	rha
**38**	Rubus	rub
**40**	Rosaceae	ros
**42**	Salix	sal
**44**	Trifolium	tri

	Pollen_type	Pollen_code
**47**	Liliaceae sl	lil
**50**	Vicia	vic
**51**	Betulaceae	bet
**52**	Fagaceae	fag

Fig. S2a: Heatmap of the pollen dataset

Methods & description

Ward dendrogram on the rows on Hellinger distances. Correlation on the columns after Hellinger transformation (Legendre & Legendre 2012).

The blue and green column on the left represent the sampling date : light green = July, green = August, light blue = September, dark blue = October.

The row names are composed of the Apiary ID and the number of he month (08 = August etc.) corresponding to the sample. We took only the 25 most abundant pollen types.

Interpretation

There are two main groups of samples (rows).

The bottom group is manly composed of samples from August (green). This group is itself composed of two groups: one group has massively exploited clover (*Trifolium*), the other group has exploited a very wide variety of pollen resources including Rosaceae, Taraxacum, Other Asteraceae, Plantaginaceae, *Trifolium*, etc.

The top group is itself divided into 3 groups characterized by the massive exploitation of Phacelia or Ivy or Brassicaceae respectively.

Fig S2a.

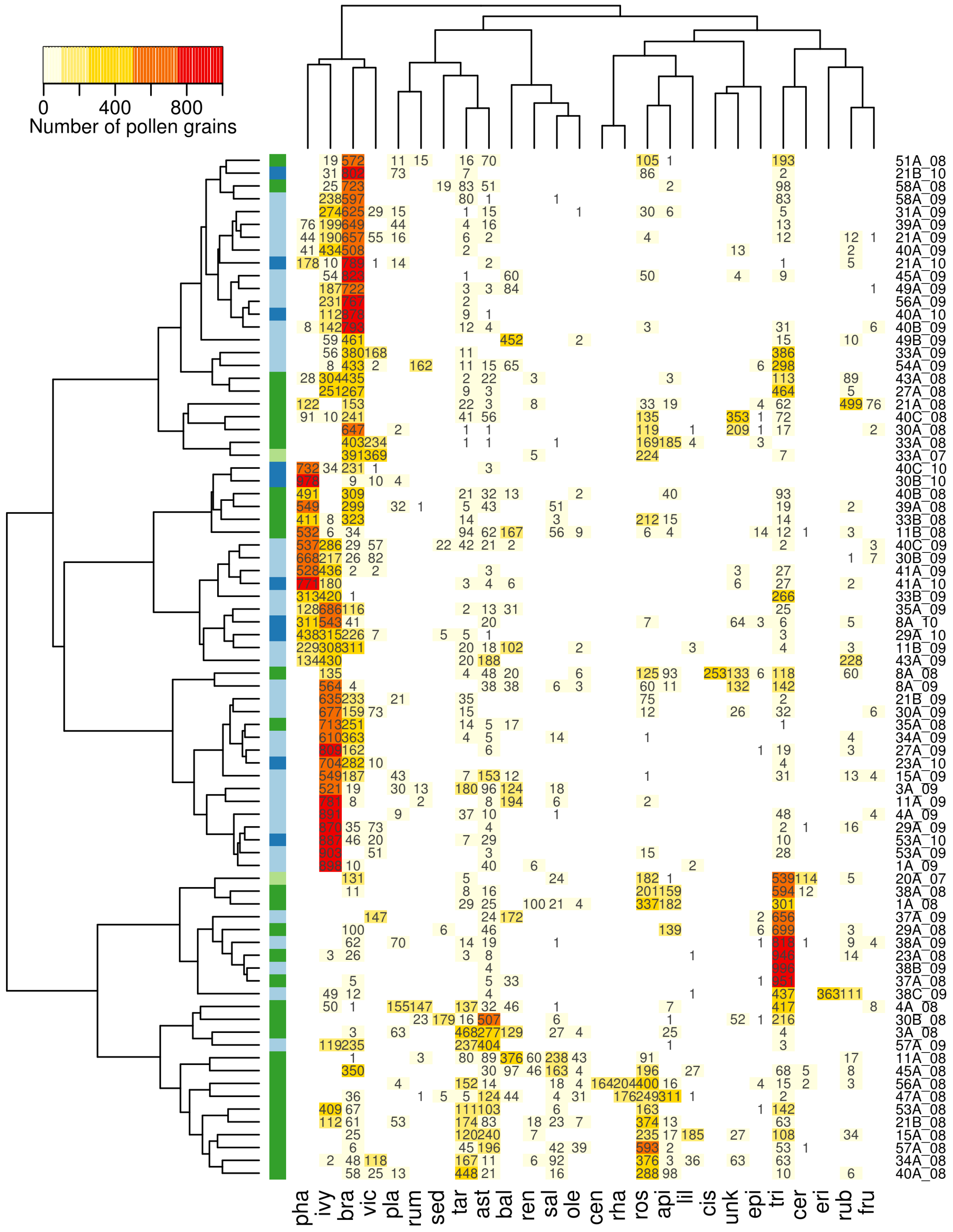
0
400
800
Number of pollen grains
pha
ivy
bra
vic
pla
rum
sed
tar
ast
bal
ren
sal
ole
cen
rha
ros
api
lil
cis
unk
epi
tri
cer
eri
rub
fru
51A_08
21B_10
58A_08
58A_09
31A_09
39A_09
21A_09
40A_09
21A_10
45A_09
49A_09
56A_09
40A_10
40B_09
49B_09
33A_09
54A_09
43A_08
27A_08
21A_08
40C_08
30A_08
33A_08
33A_07
40C_10
30B_10
40B_08
39A_08
33B_08
11B_08
40C_09
30B_09
41A_09
41A_10
33B_09
35A_09
8A_10
29A_10
11B_09
43A_09
8A_08
8A_09
21B_09
30A_09
35A_08
34A_09
27A_09
23A_10
15A_09
3A_09
11A_09
4A_09
29A_09
53A_10
53A_09
1A_09
20A_07
38A_08
1A_08
37A_09
29A_08
38A_09
23A_08
38B_09
37A_08
38C_09
4A_08
30B_08
3A_08
57A_09
11A_08
45A_08
56A_08
47A_08
53A_08
21B_08
15A_08
57A_08
34A_08
40A_08

es

Fig. S2b: Total number of pollen grains from different botanical origin per month

The two samples form end July are not shown but have a composition very similar to the August samples (see Supplementary 2 at https://figshare.com/s/86785808b5709331aa1c)

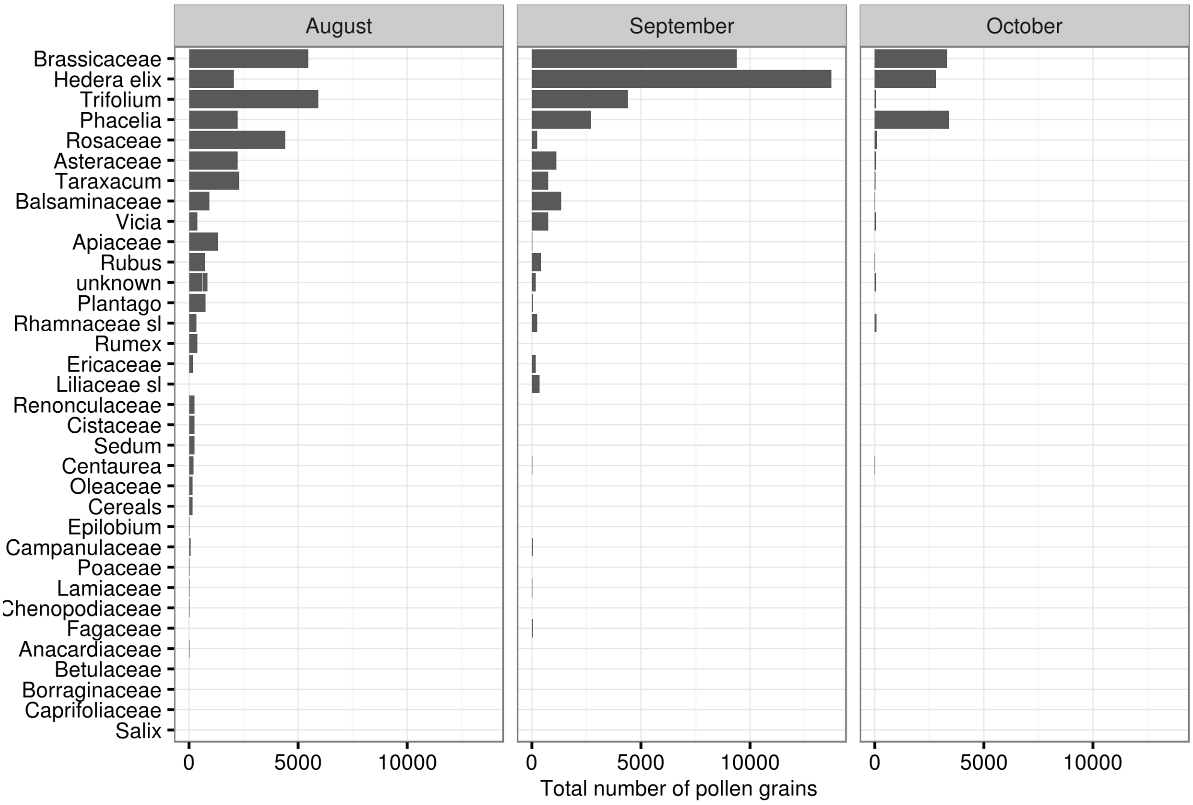

Pesticides vs Agricultural Landscape

Boscalid

Table S4: Boscalid vs cultures models

ID	Product	Buffer	Variable	AICc	AICc. delta	AICc. w	AUC	slope	LRT	p
1	bosc	3000	Beet	40.01	0	0.604	0.894	0.33	29.14	0
2	bosc	3000	AllCrops	42.81	2.798	0.149	0.894	0.18	26.34	0
3	bosc	3000	AuthorizedCrops	44.76	4.75	0.056	0.883	0.19	24.39	0
4	bosc	3000	Potato	45.24	5.231	0.044	0.92	0.36	23.9	0
5	bosc	3000	Cereals	45.74	5.734	0.034	0.863	0.21	23.4	0
6	bosc	1000	AllCrops	46.06	6.054	0.029	0.846	0.47	23.08	0
7	bosc	1500	AllCrops	46.4	6.386	0.025	0.851	0.31	22.75	0
8	bosc	1500	AuthorizedCrops	47.03	7.016	0.018	0.854	0.34	22.12	0
9	bosc	1000	AuthorizedCrops	47.35	7.344	0.015	0.84	0.53	21.79	0
10	bosc	500	AllCrops	48.79	8.779	0.007	0.834	0.7	20.36	1E-05
11	bosc	1500	Beet	50.08	10.07	0.004	0.823	0.43	19.07	1E-05
12	bosc	1500	Cereals	50.36	10.35	0.003	0.837	0.34	18.78	1E-05
13	bosc	1000	Cereals	50.49	10.48	0.003	0.817	0.51	18.66	2E-05
14	bosc	500	AuthorizedCrops	51.44	11.43	0.002	0.806	0.63	17.71	3E-05
15	bosc	3000	Fruits	52.22	12.21	0.001	0.713	0.62	16.92	4E-05
16	bosc	1000	Beet	53.35	13.34	0.001	0.746	0.5	15.79	7E-05
17	bosc	500	Cereals	54.19	14.18	0.001	0.817	0.7	14.95	0.00011
18	bosc	3000	Vegetables	54.63	14.62	0	0.873	0.4	14.52	0.00014
19	bosc	1000	Flax	54.76	14.75	0	0.779	0.83	14.38	0.00015
20	bosc	1500	Flax	55.25	15.24	0	0.744	0.61	13.89	0.00019
21	bosc	1500	Potato	55.61	15.6	0	0.831	0.38	13.54	0.00023
22	bosc	1000	Fabaceae	56.08	16.07	0	0.701	0.99	13.06	3E-04
23	bosc	3000	Fabaceae	56.65	16.64	0	0.776	0.51	12.5	0.00041
24	bosc	3000	Flax	57.28	17.27	0	0.76	0.34	11.87	0.00057
25	bosc	1500	Fruits	58.88	18.87	0	0.671	0.74	10.27	0.00136
26	bosc	1000	Potato	59.44	19.43	0	0.761	0.42	9.7	0.00184
27	bosc	500	Potato	59.95	19.94	0	0.703	0.7	9.19	0.00243
28	bosc	1500	Vegetables	60.04	20.03	0	0.723	0.59	9.1	0.00255
29	bosc	500	Beet	60.11	20.11	0	0.721	0.63	9.03	0.00266
30	bosc	1500	Fabaceae	60.32	20.31	0	0.729	0.57	8.83	0.00296
31	bosc	500	Flax	61.54	21.53	0	0.623	1.32	7.6	0.00583
32	bosc	500	Fabaceae	61.98	21.97	0	0.654	1.75	7.16	0.00745
33	bosc	1000	Fruits	62.07	22.06	0	0.637	0.73	7.08	0.0078

ID	Product	Buffer	Variable	AICc	AICc. delta	AICc. w	AUC	slope	LRT	p
34	bosc	1500	Grassland	62.12	22.11	0	0.709	-0.28	7.03	0.00801
35	bosc	1000	Vegetables	62.4	22.39	0	0.686	0.84	6.74	0.00941
36	bosc	3000	Grassland	62.97	22.96	0	0.669	-0.15	6.18	0.01294
37	bosc	1500	Horticulture	63.72	23.71	0	0.609	1.47	5.43	0.0198
38	bosc	1000	Grassland	65.35	25.34	0	0.629	-0.25	3.8	0.05138
39	bosc	500	Fruits	65.68	25.67	0	0.551	0.51	3.47	0.06255
40	bosc	3000	Corn	66.64	26.63	0	0.577	0.14	2.5	0.1138
41	bosc	500	Grassland	66.78	26.77	0	0.606	-0.27	2.37	0.1237
42	bosc	NA	NULL MODEL	66.92	26.91	0	0.5	NA	NA	NA
43	bosc	500	Corn	67.23	27.23	0	0.684	0.33	1.91	0.1669
44	bosc	1000	Corn	67.24	27.23	0	0.653	0.22	1.9	0.168
45	bosc	500	Cover	67.86	27.84	0	0.626	0.62	1.29	0.2561
46	bosc	1500	Corn	67.91	27.9	0	0.643	0.14	1.24	0.2657
47	bosc	3000	Cover	68.17	28.16	0	0.617	0.2	0.97	0.324
48	bosc	3000	Horticulture	68.29	28.28	0	0.577	0.36	0.86	0.3548
49	bosc	3000	Rapeseed	68.29	28.28	0	0.589	0.09	0.85	0.3562
50	bosc	1000	Cover	68.3	28.29	0	0.666	0.31	0.85	0.3566
51	bosc	500	Rapeseed	68.5	28.49	0	0.544	-0.78	0.65	0.4208
52	bosc	1500	Cover	68.65	28.64	0	0.674	0.21	0.5	0.4809
53	bosc	500	Vegetables	68.94	28.93	0	0.514	0.25	0.21	0.6475
54	bosc	1500	Rapeseed	69.09	29.08	0	0.573	-0.04	0.06	0.8085
55	bosc	1000	Rapeseed	69.14	29.14	0	0.476	0	0	0.995

Pyrimethanil

Table S5: Pyrimethanil vs cultures models

ID	Product	Buffer	Variable	AICc	AICc. delta	AICc. w	AUC	slope	LRT	p
1	pyri	3000	Rapeseed	42.18	0	0.287	0.735	0.39	8.55	0.00346
2	pyri	1000	Rapeseed	43.69	1.515	0.134	0.756	0.61	7.03	0.00801
3	pyri	1500	Rapeseed	44.25	2.074	0.102	0.731	0.45	6.47	0.01095
4	pyri	3000	Flax	46.15	3.97	0.039	0.793	0.24	4.58	0.0324
5	pyri	3000	Horticulture	46.82	4.64	0.028	0.641	-1.93	3.91	0.04807
6	pyri	500	Beet	47.09	4.908	0.025	0.683	0.47	3.64	0.05643
7	pyri	3000	Cover	47.36	5.178	0.022	0.63	0.48	3.37	0.06643
8	pyri	500	Potato	47.36	5.184	0.021	0.659	0.46	3.36	0.06666
9	pyri	1000	Fabaceae	47.66	5.482	0.018	0.619	0.52	3.07	0.07999
10	pyri	3000	Cereals	47.78	5.599	0.017	0.719	0.07	2.95	0.086
11	pyri	1000	Beet	48.11	5.93	0.015	0.65	0.22	2.62	0.1057
12	pyri	3000	All Crops	48.33	6.154	0.013	0.696	0.05	2.39	0.1218
13	pyri	NA	NULL MODEL	48.5	6.322	0.012	0.5	NA	NA	NA
14	pyri	500	All Crops	48.52	6.341	0.012	0.674	0.22	2.21	0.1374
15	pyri	1500	Fabaceae	48.59	6.415	0.012	0.544	0.32	2.13	0.1442
16	pyri	3000	Fruits	48.61	6.433	0.011	0.676	0.21	2.11	0.1459
17	pyri	1500	Fruits	48.95	6.769	0.01	0.596	0.26	1.78	0.1823
18	pyri	1500	Beet	49.03	6.853	0.009	0.626	0.13	1.69	0.193
19	pyri	3000	Beet	49.09	6.913	0.009	0.67	0.07	1.63	0.2011
20	pyri	1000	Potato	49.15	6.971	0.009	0.604	0.19	1.58	0.2093
21	pyri	1000	Fruits	49.42	7.242	0.008	0.596	0.25	1.3	0.2533
22	pyri	1000	Authorized Crops	49.42	7.243	0.008	0.596	0.25	1.3	0.2535
23	pyri	1500	Horticulture	49.47	7.291	0.007	0.537	-1.3	1.26	0.2623
24	pyri	3000	Authorized Crops	49.54	7.36	0.007	0.617	0.17	1.19	0.2758
25	pyri	1000	Grassland	49.56	7.38	0.007	0.646	-0.17	1.17	0.2799
26	pyri	1500	Authorized Crops	49.58	7.405	0.007	0.563	0.22	1.14	0.2851
27	pyri	500	Flax	49.66	7.484	0.007	0.569	0.49	1.06	0.3024
28	pyri	500	Fabaceae	49.71	7.533	0.007	0.533	-1.07	1.01	0.3138
29	pyri	1000	Cover	49.73	7.551	0.007	0.628	0.41	1	0.3183
30	pyri	500	Authorized Crops	49.74	7.557	0.007	0.594	0.24	0.99	0.3196
31	pyri	500	Fruits	49.74	7.56	0.007	0.594	0.24	0.99	0.3204

ID	Product	Buffer	Variable	AICc	AICc. delta	AICc. w	AUC	slope	LRT	p
32	pyri	3000	Fabaceae	49.81	7.628	0.006	0.583	0.15	0.92	0.3377
33	pyri	500	Vegetables	49.95	7.775	0.006	0.533	-6.17	0.77	0.3797
34	pyri	1500	Cover	50	7.82	0.006	0.593	0.31	0.73	0.394
35	pyri	1000	All Crops	50.01	7.831	0.006	0.587	0.07	0.72	0.3973
36	pyri	1000	Flax	50.02	7.842	0.006	0.609	0.2	0.71	0.4011
37	pyri	500	Cereals	50.08	7.896	0.006	0.644	0.15	0.65	0.4197
38	pyri	500	Grassland	50.08	7.901	0.006	0.581	-0.17	0.65	0.4213
39	pyri	3000	Potato	50.19	8.013	0.005	0.619	0.05	0.53	0.4649
40	pyri	1500	Flax	50.24	8.057	0.005	0.576	0.13	0.49	0.4837
41	pyri	1500	Vegetables	50.24	8.059	0.005	0.554	-0.19	0.49	0.4847
42	pyri	1500	All Crops	50.28	8.101	0.005	0.57	0.04	0.45	0.5043
43	pyri	500	Rapeseed	50.37	8.189	0.005	0.63	0.54	0.36	0.5496
44	pyri	1500	Corn	50.39	8.207	0.005	0.6	-0.09	0.34	0.5595
45	pyri	3000	Grassland	50.39	8.208	0.005	0.589	-0.04	0.34	0.5603
46	pyri	1000	Corn	50.44	8.26	0.005	0.598	-0.11	0.29	0.5919
47	pyri	1500	Cereals	50.53	8.347	0.004	0.574	0.03	0.2	0.6542
48	pyri	1000	Cereals	50.62	8.443	0.004	0.559	0.03	0.1	0.747
49	pyri	3000	Vegetables	50.63	8.454	0.004	0.522	0.04	0.09	0.7602
50	pyri	1000	Vegetables	50.63	8.456	0.004	0.541	-0.12	0.09	0.7629
51	pyri	500	Cover	50.68	8.497	0.004	0.52	0.15	0.05	0.8223
52	pyri	1500	Grassland	50.68	8.497	0.004	0.541	-0.03	0.05	0.8227
53	pyri	3000	Corn	50.68	8.498	0.004	0.552	-0.02	0.05	0.8237
54	pyri	500	Corn	50.68	8.505	0.004	0.474	0.06	0.04	0.8368
55	pyri	1500	Potato	50.7	8.523	0.004	0.513	0.02	0.02	0.8758

Table S6: Pyrimethanil vs peas crops models
Here we examine the hypothesis that pyrimethanil treatments of peas could explain pyrimethanil contaminations (in the previous analysis, peas are in the grouped culture Fabaceae with other crops from this family) The peas models performs better than the null model and that the previous Fabaceae models however these models are not very good (difference of AICc low and AUC low).

ID	Product	Buffer	Variable	AICc	AICc. delta	AICc. w	AUC	slope	LRT	p
1	pyri	1500	peas	44.9	0	0.436	0.665	0.51	5.82	0.01582
2	pyri	1000	peas	45.66	0.752	0.299	0.676	0.66	5.07	0.02433
3	pyri	3000	peas	46.85	1.942	0.165	0.739	0.27	3.88	0.04884
4	pyri	NA	NULL MODEL	48.5	3.598	0.072	0.5	NA	NA	NA
5	pyri	500	peas	50.37	5.47	0.028	0.481	-0.61	0.35	0.5526

Dimethoate

Table S7: Dimethoate vs cultures models

ID	Product	Buffer	Variable	AICc	AICc. delta	AICc. w	AUC	slope	LRT	p
1	dime	1000	Cereals	16.52	0	0.989	0.991	3.32	37.55	0
2	dime	1000	AllCrops	26.93	10.4	0.005	0.955	0.93	27.15	0
3	dime	1500	Beet	29.61	13.08	0.001	0.942	0.77	24.47	0
4	dime	1000	Beet	30.29	13.76	0.001	0.946	0.97	23.79	0
5	dime	1500	Cereals	30.68	14.15	0.001	0.906	0.62	23.4	0
6	dime	1500	AuthorizedCrops	31.37	14.85	0.001	0.92	0.67	22.7	0
7	dime	1500	AllCrops	31.4	14.88	0.001	0.929	0.46	22.67	0
8	dime	1000	AuthorizedCrops	33.26	16.73	0	0.924	0.72	20.82	1E-05
9	dime	3000	Beet	33.73	17.21	0	0.915	0.36	20.34	1E-05
10	dime	3000	AuthorizedCrops	34.36	17.84	0	0.924	0.3	19.71	1E-05
11	dime	500	Beet	36.15	19.62	0	0.897	1.24	17.93	2E-05
12	dime	1000	Vegetables	36.19	19.67	0	0.761	1.67	17.88	2E-05
13	dime	3000	AllCrops	37.03	20.51	0	0.902	0.17	17.04	4E-05
14	dime	3000	Cereals	37.3	20.78	0	0.884	0.22	16.77	4E-05
15	dime	500	Cereals	40.09	23.57	0	0.911	1.02	13.98	0.00018
16	dime	500	AllCrops	41.03	24.5	0	0.902	0.67	13.04	3E-04
17	dime	3000	Potato	41.3	24.78	0	0.871	0.3	12.77	0.00035
18	dime	3000	Vegetables	41.5	24.97	0	0.888	0.4	12.58	0.00039
19	dime	1500	Vegetables	42.76	26.24	0	0.79	0.71	11.31	0.00077
20	dime	1500	Flax	43.45	26.92	0	0.772	0.58	10.63	0.00111
21	dime	500	AuthorizedCrops	43.71	27.19	0	0.866	0.62	10.36	0.00129
22	dime	1000	Flax	44.21	27.69	0	0.79	0.74	9.86	0.00169
23	dime	1500	Grassland	46.11	29.59	0	0.741	-0.39	7.96	0.00477
24	dime	3000	Flax	46.35	29.82	0	0.812	0.32	7.73	0.00544
25	dime	500	Vegetables	46.68	30.16	0	0.643	7.45	7.39	0.00655
26	dime	1500	Horticulture	47.1	30.57	0	0.754	1.69	6.98	0.00825
27	dime	1500	Potato	47.52	31	0	0.792	0.3	6.55	0.01048
28	dime	1000	Potato	47.59	31.06	0	0.783	0.39	6.49	0.01088
29	dime	3000	Grassland	47.72	31.2	0	0.723	-0.21	6.35	0.01172
30	dime	3000	Fabaceae	48.09	31.57	0	0.712	0.43	5.98	0.01443
31	dime	500	Potato	48.65	32.12	0	0.826	0.58	5.43	0.01984
32	dime	1000	Fabaceae	49.23	32.71	0	0.618	0.65	4.84	0.02778

ID	Product	Buffer	Variable	AICc	AICc. delta	AICc. w	AUC	slope	LRT	p
33	dime	1000	Grassland	49.76	33.24	0	0.712	-0.33	4.31	0.03784
34	dime	3000	Fruits	49.94	33.41	0	0.654	0.29	4.14	0.04191
35	dime	500	Rapeseed	50.34	33.81	0	0.578	-108.4	3.74	0.05327
36	dime	3000	Horticulture	50.45	33.93	0	0.67	0.84	3.62	0.05701
37	dime	500	Flax	50.75	34.22	0	0.679	0.83	3.33	0.06818
38	dime	NA	NULL MODEL	51.85	35.32	0	0.5	NA	NA	NA
39	dime	500	Fabaceae	52.41	35.89	0	0.576	0.72	1.66	0.1972
40	dime	1500	Fabaceae	52.77	36.24	0	0.585	0.25	1.31	0.2525
41	dime	500	Fruits	52.98	36.46	0	0.464	-1.07	1.09	0.296
42	dime	500	Grassland	53.38	36.85	0	0.638	-0.18	0.7	0.4031
43	dime	500	Cover	53.53	37.01	0	0.58	0.47	0.54	0.4625
44	dime	500	Corn	53.55	37.03	0	0.618	0.21	0.52	0.4696
45	dime	3000	Rapeseed	53.68	37.15	0	0.551	0.07	0.4	0.5287
46	dime	3000	Corn	53.77	37.25	0	0.58	0.06	0.3	0.584
47	dime	1500	Rapeseed	53.78	37.26	0	0.498	0.09	0.29	0.5903
48	dime	1000	Fruits	53.83	37.31	0	0.446	-0.15	0.24	0.6221
49	dime	1000	Cover	53.93	37.4	0	0.469	-0.16	0.15	0.7023
50	dime	3000	Cover	53.97	37.45	0	0.562	0.08	0.1	0.7459
51	dime	1000	Rapeseed	54.06	37.54	0	0.565	-0.03	0.01	0.9088
52	dime	1500	Fruits	54.06	37.54	0	0.556	0.03	0.01	0.9094
53	dime	1500	Corn	54.07	37.55	0	0.518	0.01	0	0.9576
54	dime	1000	Corn	54.07	37.55	0	0.475	-0.01	0	0.9581
55	dime	1500	Cover	54.07	37.55	0	0.589	0.01	0	0.9698

Pollen vs Landscape

Fig. S3 Pollen vs landscape RDA triplot

Methods

We performed a redundancy analysis (RDA) to see if the matrix of pollen type can be explained by the matrix of agricultural land use areas. This is equivalent to a multiple regression with more than one dependent y variable. This method also uses PCA approaches to obtain a visual summary of the results (triplots).

We performed a Hellinger transformation on the pollen data (to avoid double 0 to be considered as similarities) and a square root transformation of the landscape areas. We worked on a standardized pollen matrix (after Hellinger transformation) and we used the 1500 m buffer data and grouped Beet Potato and cereals areas to lessen multicollinearity problems. We used the 10 most abundant pollen types to simplify the plot.

Interpretation

The adjusted R squared shows that the landscape data explains only ~9% of the pollen data variance (27% for unadjusted R^2). The correlation between pollen type is very low so it is difficult to find a matrix that explains most of the variance.

```
## $r.squared
## [1] 0.2685294
##
## $adj.r.squared
## [1] 0.09361248
```

In the following plot you can interpret the angles between the pollen (red lines) and land use areas (blue arrows) as correlation. The first two axes explain 56% of the constrained variance (58% * 9% of the total variance) So this plan is a correct representation of the variance explained by the land use areas.

The triplot suggests the following correlations :

- a positive relationship between Vicia pollen and BeetCeralsPotato and Vegetables
- a positive relationship between Trifolium pollen and Grasslands
- a positive relationship between Rosaceae, taraxacum pollen and Fruits cultures and horticulture
- a negative relationship between the last 2 groups
- a negative relationship between Asteraceae pollen and Rapeseed

NB : RDA is a simplified representation of complex dataset. Some relationship might be hidden in other dimensions.

Fig S3: Pollen vs landscape RDA triplot

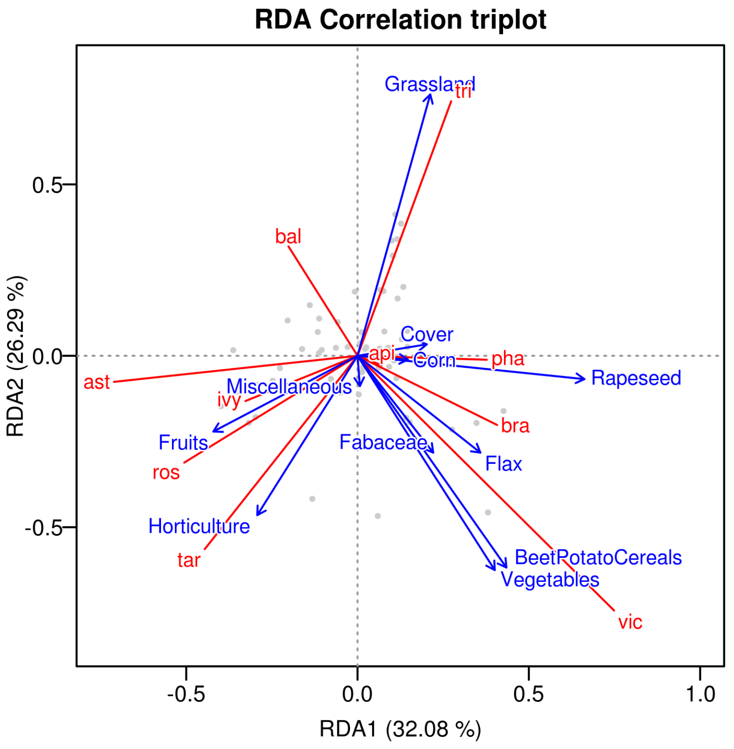

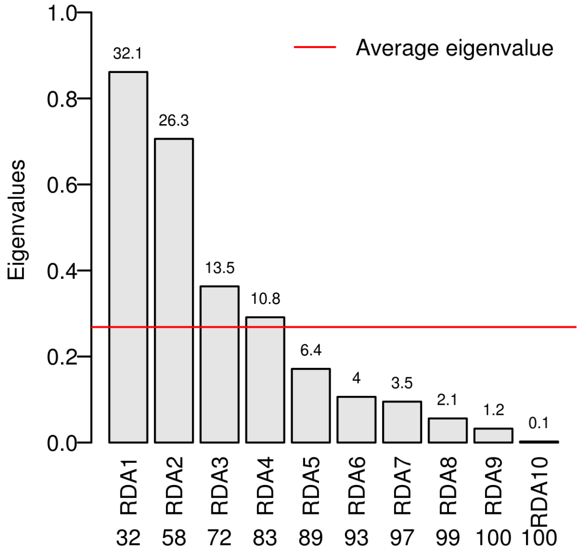

Supplement: Supplementary file 1 — Supplementary information [file 41598_2017_3467_MOESM1_ESM.pdf]
